# Acquired Autoimmune Factor XIII Deficiency as a Novel Immune‐Related Adverse Event of Combined Ipilimumab and Nivolumab Therapy for Renal Cell Carcinoma: A Case Report

**DOI:** 10.1002/iju5.70207

**Published:** 2026-07-08

**Authors:** Shigeyasu Tetsushi, Koura Mikifumi, Ootsubo Satoshi, Hasegawa Shuji, Tatsugami Katsunori

**Affiliations:** ^1^ Kitakyushu Shiritsu Iryo Center Kitakyushu Japan

**Keywords:** acquired autoimmune factor XIII deficiency, immune checkpoint inhibitors (ICIs), immune‐related adverse events (irAEs), ipilimumab, nivolumab, renal cell carcinoma

## Abstract

**Introduction:**

Combination therapy with immune checkpoint inhibitors (ICIs) has become the standard pharmacological treatment for renal cell carcinoma; however, careful attention must be paid to immune‐related adverse events (irAEs). We report a rare case of acquired autoimmune factor XIII deficiency following ipilimumab plus nivolumab therapy.

**Case Presentation:**

A 75‐year‐old woman who had previously undergone nephrectomy for left renal cell carcinoma developed pancreatic and pulmonary metastases and was started on combination immunotherapy. Six months after treatment initiation, she presented with bleeding symptoms. Laboratory testing revealed anti‐factor XIII autoantibodies, confirming a diagnosis of acquired autoimmune factor XIII deficiency.

**Conclusion:**

To our knowledge, few reports have described this condition as an irAE. Nonetheless, other irAE‐related acquired coagulation factor deficiencies—such as factor V deficiency and acquired hemophilia A—have been reported. These precedents suggest a high likelihood that the current case represents a novel manifestation of irAE.

## Keynote Message

- We encountered a rare case of acquired autoimmune factor XIII deficiency following ipilimumab plus nivolumab therapy.
- Acquired autoimmune factor XIII deficiency is a potentially fatal condition that requires careful attention.
- In patients who develop recurrent bleeding symptoms during treatment with immune checkpoint inhibitors (ICIs), we should consider the possibility of acquired autoimmune factor XIII deficiency, even in the absence of anemia, thrombocytopenia, or abnormal prothrombin time/activated partial thromboplastin time.

## 1 Introduction

Combination therapy with immune checkpoint inhibitors (ICIs) has become the standard treatment for renal cell carcinoma. However, physicians need to pay attention to immune‐related adverse events (irAEs), which can cause serious issues in patients. IrAEs are common adverse events caused by ICIs, which overactivate immune cells and cause them to attack normal tissues, resulting in a variety of symptoms throughout the body. Although immune thrombocytopenia and hemolytic anemia are the most common hematologic irAEs, coagulation factor deficiencies have rarely been reported [1]. In reports on coagulation factor deficiencies, acquired hemophilia A (caused by autoantibodies against factor VIII) and factor V deficiency have been reported [2]. However, to the best of our knowledge, acquired autoimmune factor XIII deficiency has not been reported. We report a rare case of acquired autoimmune factor XIII deficiency following ipilimumab plus nivolumab combination therapy.

## 2 Case Presentation

A 75‐year‐old female patient had no significant family or past medical history. In X‐16 years, she underwent radical nephrectomy for left renal cell carcinoma. The pathological diagnosis was clear cell renal cell carcinoma, G2, pT3a. Interferon was administered as adjuvant therapy but was discontinued because of general fatigue. In X‐3 years, she underwent a biopsy because a computed tomography scan showed a mass lesion in the head of the pancreas. The diagnosis was pancreatic metastasis of clear cell renal cell carcinoma. The patient underwent partial pancreatectomy. The pathological diagnosis was metastasis of clear cell renal cell carcinoma. Three years after surgery, a computed tomography scan also showed an enlarged mass on the residual pancreas, which was diagnosed as pancreatic metastasis of renal carcinoma. Using International Metastatic Renal Cell Carcinoma Database Consortium (IMDC) risk criteria, the patient was classified as intermediate risk because of anemia (hemoglobin concentration: 11.7 g/dL [117 g/L]) and received ipilimumab + nivolumab combination therapy.

In October X years, the patient had low thyroxine and high blood glucose concentrations after two administrations of ipilimumab and nivolumab. The diagnosis was type 1 diabetes mellitus and hypothyroidism due to irAEs. One month after treatment for irAEs, the patient's condition improved and nivolumab was resumed.

Six months after the initiation of treatment, disease progression was observed due to increased pancreatic metastases and the appearance of lung metastases. Two months after starting axitinib therapy, the patient developed bleeding symptoms accompanied by anemia and lower‐extremity swelling due to hematoma formation (Figure 1). Despite discontinuation of axitinib, the bleeding symptoms persisted. Laboratory evaluation of the coagulation system revealed a platelet count of 25 × 10^4^/μL. Prothrombin time (PT) was 98.9%, the prothrombin time–international normalized ratio (PT‐INR) was 1.00, activated partial thromboplastin time (APTT) was 22.4 s, fibrinogen level was 352 mg/dL, and antithrombin III (AT‐III) activity was 97%, all of which were within normal ranges. In contrast, factor XIII activity was markedly reduced to 10%, and factor XIII autoantibodies were detected, leading to a diagnosis of acquired autoimmune factor XIII deficiency. At the time of diagnosis, the hemoglobin level was 7 g/dL (70 g/L), and purpura was observed on the lower extremities.

**FIGURE 1:**
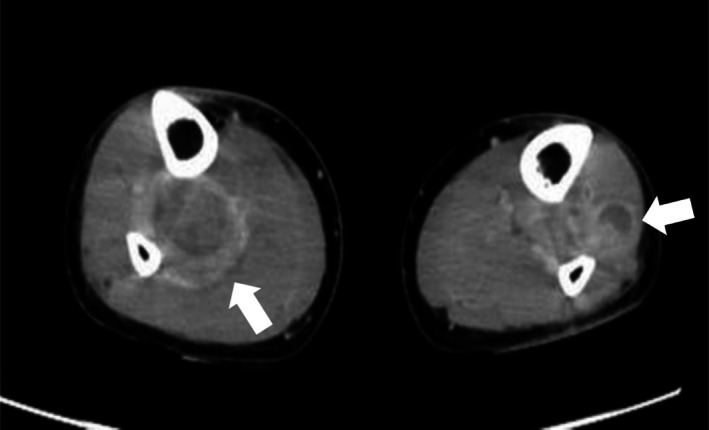
CT scan of the lower legs revealing intramuscular hematomas (white arrows) that developed after discontinuation of axitinib treatment.

Treatment with prednisolone (1.0 mg/kg/day) and fibrogammin (50 U/kg/day) was started, and the purpura gradually resolved. One month after this treatment, hemoglobin concentration improved to 12 g/dL (120 g/L) and the purpura had disappeared (Figure 2). After restarting axitinib treatment, no exacerbation of bleeding symptoms was noted.

**FIGURE 2:**
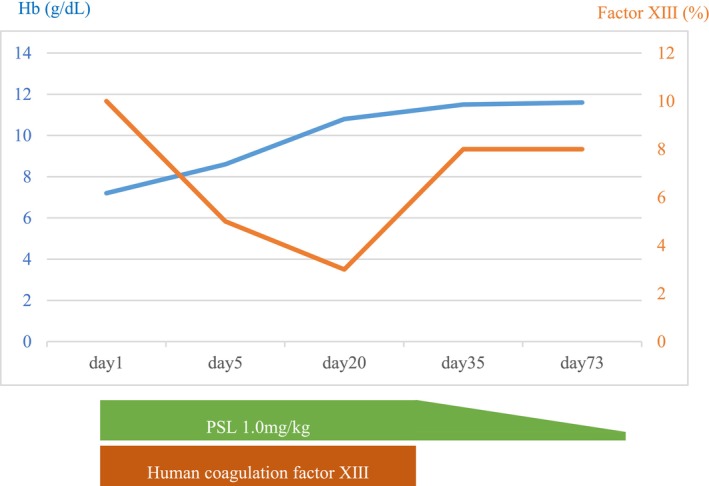
Clinical course showing the kinetics of hemoglobin levels and factor XIII activity during treatment with prednisolone and human coagulation factor XIII concentrate.

## 3 Conclusion

Acquired autoimmune factor XIII deficiency is a disease characterized by the acquired appearance of autoantibodies against factor XIII. These autoantibodies result in severely reduced factor XIII activity and sudden onset of bleeding symptoms, although the prothrombin time and activated partial thromboplastin time are within reference limits [3]. The cause of acquired autoimmune factor XIII deficiency can be due to a variety of underlying diseases and conditions, including autoimmune diseases, malignancies, infections, and pregnancy/delivery, but approximately half of the cases are idiopathic [4]. Symptoms include muscle and subcutaneous hemorrhage, and in some cases, sudden death may occur depending on the bleeding site, such as intracranial, intrathoracic, intraperitoneal, or retroperitoneal hemorrhage [5]. The diagnostic criteria for acquired autoimmune factor XIII deficiency are shown in Table 1 [6].

**TABLE 1:** Diagnostic criteria of autoimmune hemorrhaphilia factor XIII.

Diagnostic criteria of autoimmune hemorrhaphilia factor XIII:
It is possible if all of the following five conditions are met:
Bleeding symptoms are generally present
2 There is no family history of congenital/hereditary factor XIII deficiency
3 There is no history of bleeding symptoms, including those related to hemostatic stress such as surgery, trauma, suture removal, or childbirth
4 Bleeding related to anticoagulant or antiplatelet agents can be ruled out
5 Laboratory tests show a decrease in factor XIII activity or antigen levels (Under 50% of the standard reference level)
Probable:
In addition to (1) through (5) above, a factor XIII inhibitor is present
Define
In addition to (1) through (5) above, anti–factor XIII autoantibodies are positive

In the present case, the patient had persistent bleeding despite normal prothrombin time (PT) and activated partial thromboplastin time (APTT), and factor XIII autoantibodies were detected. Although reports suggesting an association between tyrosine kinase inhibitors (TKIs), including axitinib, and factor XIII deficiency are extremely limited, a causal relationship is considered unlikely.

On the other hand, ICIs have been reported to be associated with various autoimmune coagulation disorders, such as acquired hemophilia A, and the development of acquired factor XIII deficiency has also been suggested as a possible immune‐related adverse event [7]. Furthermore, the absence of worsening bleeding after reintroduction of axitinib supports a limited role for TKIs in the pathogenesis of this condition.

Based on these findings, the present case was considered to represent acquired autoimmune factor XIII deficiency associated with immune checkpoint inhibitor therapy.

Furthermore, the patient had no clinical features suggestive of other autoimmune diseases, and no new medications other than axitinib had been introduced. Therefore, these factors were considered unlikely to have contributed to the development of acquired factor XIII deficiency. Although the malignancy itself cannot be excluded as a potential cause of this condition, the tumor had been diagnosed prior to the initiation of nivolumab and ipilimumab therapy. In addition, the improvement in bleeding symptoms following discontinuation of ICI therapy more strongly supports the involvement of immunotherapy in the pathogenesis of this condition.

Treatment of acquired autoimmune factor XIII deficiency includes hemostatic therapy and antibody eradication/elimination therapy [8]. Factor XIII concentrates should be administered intravenously to achieve immediate hemostasis and continued until bleeding is controlled because factor XIII preparations rapidly lose efficacy owing to inhibitor antibodies and increased immune complex elimination [9]. Corticosteroids and immunosuppressive drugs, such as cyclophosphamide, are often effective in antibody eradication/elimination therapy. Rituximab, cyclosporine A, and azathioprine should also be considered for treatment‐resistant cases [8]. The prognosis for acquired autoimmune factor XIII deficiency is poor, with 30% of patients dying from the disease, 40% requiring continuous treatment, and 30% achieving remission [10]. In this case, the patient's symptoms were alleviated with fibrogammin and steroids, and the patient has remained recurrence‐free while receiving prednisolone at 3 mg/day.

In conclusion, for patients with recurrent bleeding symptoms during treatment with ICIs, despite the absence of anemia, thrombocytopenia, and an abnormal prothrombin time/activated partial thromboplastin time, physicians should consider the possibility of acquired autoimmune factor XIII deficiency.

## Ethics Statement

This single‐patient case report is exempt under our institutional policy.

## Consent

Written informed consent for publication, including images, was obtained from the patient.

## Conflicts of Interest

The authors declare no conflicts of interest.

## Data Availability

Data sharing not applicable to this article as no datasets were generated or analyzed during the current study.
